# Transpulmonary pressure monitoring in critically ill patients: pros and cons

**DOI:** 10.1186/s13054-024-04950-y

**Published:** 2024-05-25

**Authors:** Lorenzo Ball, Daniel Talmor, Paolo Pelosi

**Affiliations:** 1 Department of Surgical Sciences and Integrated Diagnostics (DISC), University of Genoa, Viale Benedetto XV 16, Genoa, Italy; 2 Anesthesia and Intensive Care, San Martino Policlinico Hospital, IRCCS for Oncology and Neurosciences, Genoa, Italy; 3 Department of Anesthesia, Critical Care, and Pain Medicine, Beth Israel Deaconess Medical Center and Harvard Medical School, Boston, MA USA

**Keywords:** ARDS, Transpulmonary pressure, Esophageal pressure

## Abstract

The use of transpulmonary pressure monitoring based on measurement of esophageal pressure has contributed importantly to the personalization of mechanical ventilation based on respiratory pathophysiology in critically ill patients. However, esophageal pressure monitoring is still underused in the clinical practice. This technique allows partitioning of the respiratory mechanics between the lungs and the chest wall, provides information on lung recruitment and risk of barotrauma, and helps titrating mechanical ventilation settings in patients with respiratory failure. In assisted ventilation modes and during non-invasive respiratory support, esophageal pressure monitoring provides important information on the inspiratory effort and work of breathing. Nonetheless, several controversies persist on technical aspects, interpretation and clinical decision-making based on values derived from this monitoring technique. The aim of this review is to summarize the physiological bases of esophageal pressure monitoring, discussing the pros and cons of its clinical applications and different interpretations in critically ill patients undergoing invasive and non-invasive respiratory support.

## Introduction

Transpulmonary pressure (P_L_) corresponds to the distending force (stress) applied to the lungs which results in their mechanical deformation (strain) [1]. Stress and strain are linked by a linear relationship in healthy subjects and in patients with acute respiratory distress syndrome (ARDS), namely $stress=k \bullet strain$, where k is specific elastance [2]. The correct physiological definition of transpulmonary pressure is ${P}_{L}={P}_{ALV}-{P}_{pl}$, where P_ALV_ is the alveolar pressure and P_pl_ is the pleural pressure. While P_ALV_ equals the airway pressure (P_AW_) under static conditions at end-inspiration or end-expiration, P_pl_ requires indirect estimation. Due to the anatomical position of the esophagus in the pleural space, esophageal pressure (P_es_) represents a surrogate of the P_pl_ [1, 3]: therefore, in the clinical practice, transpulmonary pressure can be estimated as ${P}_{L}={P}_{AW}-{P}_{es}$. The use of such approximation has contributed importantly to the knowledge of the respiratory pathophysiology in critically ill patients and individualization of mechanical ventilation [4]. The use of esophageal balloons to measure P_es_ requires expertise and the correct interpretation of P_es_-derived transpulmonary pressure warrants deep understanding of the assumptions underlying the use of P_es_ as an estimate of P_pl_. Possibly as a consequence of this complexity, esophageal pressure monitoring is still underused in the clinical practice [1] and less than 1% of patients with ARDS received this monitoring tool in a recent large international observational study [5].

The aim of this review is to summarize the physiological bases of esophageal pressure monitoring, discussing the pros and cons of its clinical applications and different interpretations in critically ill patients undergoing non-invasive and invasive respiratory support.

## Determinants of esophageal pressure

In the upright position, esophageal pressure changes reflect accurately the overall changes occurring in pleural pressure applied to the lungs’ surface at a specific site [6]. However, in the supine position, several factors may influence the value of pressure measured inside the esophagus using an air-filled balloon. Among them, the most important determinants of P_es_ are the following: chest wall elastance, the height of the chest wall, the distension of the abdomen pushing the diaphragm upwards and the weight of mediastinal organs lying above the esophageal balloon [7]. Moreover, the elastance of the esophageal wall, the reaction of smooth musculature to the presence of the balloon and the elastance of the esophageal balloon itself affect the measurement, while the transmission of cardiac contractions introduces artifacts which may further complicate the interpretation of P_pl_.

Despite these known limitations, correct placement of the device allows an acceptable estimation of P_pl_ changes also in the supine position, with a good correlation with the pressure measured directly in the middle pleural space shown in experimental studies [8, 9]. Changes in body postures have been applied in a study in healthy subjects to estimate the influence of mediastinal and lung weight on P_es_, which resulted in a mean of 3 cmH_2_O [10]. If not accounted for, this additional pressure results in slight overestimation of the P_pl_, thus underestimation of the P_L_, in the dependent lung regions and in slight underestimation of the P_pl_, thus overestimation of the P_L_, in the most non-dependent lung regions. Moreover, esophageal balloons are often placed in patients requiring enteral feeding, however, the presence of a nasogastric tube does not alter significantly the measurement of P_es_ [11], and the industry has made available catheters combining the function of a nasogastric tube and an esophageal pressure probe [12].

Use of P_es_ as a surrogate for P_pl_ **Pros:** The absolute value of P_es_ represents a reasonable surrogate of the P_pl_ and allows a pragmatic estimation of transpulmonary pressure at the bedside.

**Cons:** The P_es_ can become different from the actual P_pl_ in case of relevant weight of the mediastinum and injured lungs, which can be difficult to assess at the bedside.

### Esophageal balloon positioning

The pressure inside the esophagus varies along its axis. Pressure is irregular in different portions of the esophagus as assessed using multi-probe high resolution manometry [13]. Nonetheless, all studies in respiratory physiology focused on measurements performed in the distal third of the esophagus: correct placement of the probe is therefore crucial. However, a study comparing middle (20–35 cm from the mouth) versus distal (40–45 cm from the mouth) esophageal probe positioning showed minimal influence on estimates of P_L_ [14], suggesting that a certain margin of flexibility can be accepted. The presence of cardiac pulse artifacts further confirms the positioning in the lower esophageal third. Certain manufacturers of esophageal balloons inserted a radio-opaque marker to allow radiological confirmation of the correct positioning [12]. In addition to correct positioning, adequate inflation volume of the probe is key to correct interpretation of esophageal pressure.

Use of standard positioning based on insertion depth **Pros:** Adequate in most patients.

**Cons:** In case of extremely short or tall patients positioning adjustments may be necessary, as is the case of subjects with anatomical variants resulting in difficult insertion. Moreover, blind positioning may cause accidental misplacement in the airway in deeply sedated patients; in this case, direct or video-laryngoscopy should be considered to confirm correct positioning.

### Esophageal balloon inflation

Most esophageal probes manufacturers suggest inflating the balloon with a fixed amount of air, in a range from 0.5 to 4 ml, according to the size and elastic properties of the device. However, technical characteristics of the balloon such as diameter, size, material and compliance of the cuff affect the transmission of pressure changes in the chest wall to the balloon according to its inflation volume [15]. Several authors suggest titrating volume inflation individually. In fact, under-filling would result in minimal cardiac artefacts [16] but under-estimation of both baseline P_es_ and P_es_ swings during tidal breathing, while over-filling would over-estimate P_es_ [17]. An optimal inflation should be aimed at maintaining the ratio of changes of the P_es_ and P_aw_ closest to 1 during an airway occlusion test [18], while other experts suggested inflating it in order to remain in the linear part of the esophageal balloon pressure–volume curve while maximizing the difference between P_es,end-inspiratory_ and P_es,end-expiratory_ [15]. Since most balloons are connected to the ventilator auxiliary port or dedicated monitoring system through a three-way stopcock and a tube, air leaks may occur: balloon filling should be checked periodically to ensure quality of measurements. To reduce transmission of cardiac noise and to minimize the risk of leaks, liquid-filling of balloons has been proposed [19], but seldom used in the clinical practice.

Use of standard balloon inflation volume **Pros:** Inflating the balloon based on the manufacturer recommendations provides acceptable measurements of P_es_ in many clinical conditions.

**Cons:** Standard inflation volume can result in over- or under-estimation of P_pl_ and individual titration could be necessary to avoid misinterpretation of P_L_.

### Occlusion maneuvers to confirm positioning and inflation

**Fig. 1:**
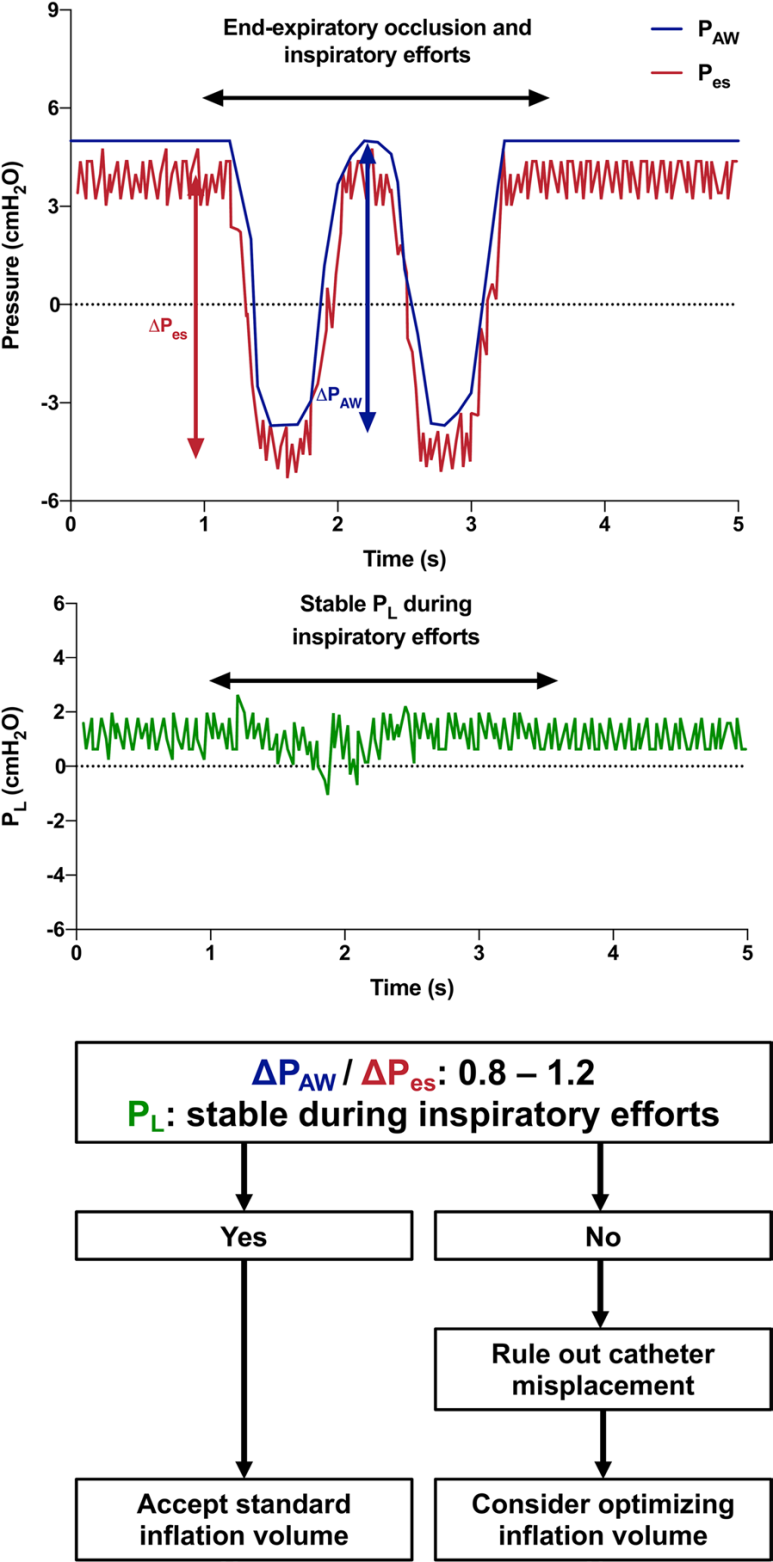
Occlusion test to confirm correct positioning and inflation of the esophageal balloon in an active patient receiving assisted ventilation. An end-expiratory occlusion is performed, during which negative deflections of the pleural (P_es_) and airway (P_AW_) pressures are observed. During inspiratory efforts, the transpulmonary pressure (P_L_) remains stable

**Fig. 2:**
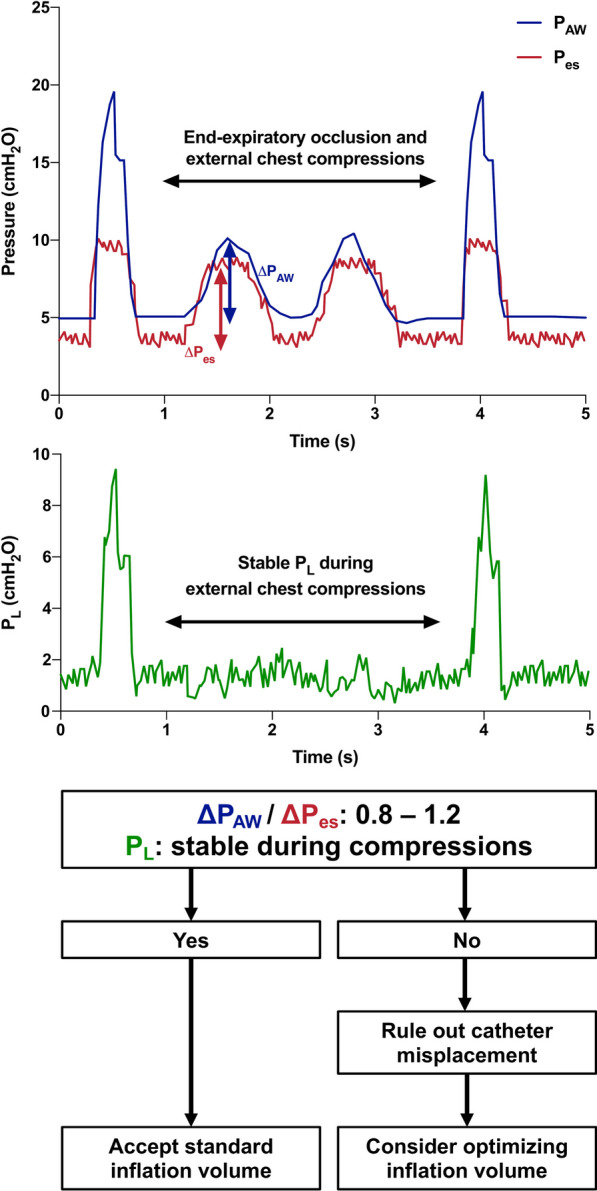
Occlusion test to confirm correct positioning and inflation of the esophageal balloon in a sedated patient without spontaneous breathing activity receiving controlled ventilation. An end-expiratory occlusion is performed, and gentle external chest compressions are delivered. Positive swings of the pleural (P_es_) and airway (P_AW_) pressures reflect the increase in intrathoracic pressure due to the external compressions. During the occlusion maneuver, the transpulmonary pressure (P_L_) remains stable

As discussed above, positioning and inflation of the balloon both influence the P_es_. Correct positioning and filling can be checked using an occlusion test: when the airway is occluded at end-expiration, changes in P_pl_ are transmitted to the airway through the lungs. During occlusion, the changes of P_es_ (ΔP_es_) equal the changes of the P_aw_ (ΔP_aw_), thus their ratio should be 1 (ΔP_es_/ ΔP_aw_ = 1) [18], assuming that P_pl_ = P_es_. A tolerance of 10% or 20% is normally considered acceptable, corresponding to ΔP_es_/ ΔP_aw_ from 0.9 to 1.1 or from 0.8 to 1.2, respectively. In spontaneously breathing patients, airway pressure changes assessed during the occlusion test correspond to the negative P_es_ swings due to the isometric inspiratory efforts (Fig. 1). In sedated passive patients, pressure changes must be induced with gentle external chest compressions (Fig. 2), sufficient to generate a safe but measurable ΔP_aw_, typically values between 5 and 15 cmH_2_O are aimed for. When ΔP_aw_ and ΔP_es_ are equal, their difference, namely the ΔP_L_, is zero. In modern ventilators and monitors able to plot the P_L_ tracing in real-time (green plots in Figs. 1 and 2), verifying that P_L_ remains constant during inspiratory efforts or chest compressions at the occlusion test further confirms the correct positioning and inflation of the balloon. Figures 1 and 2 illustrate these concepts and propose an algorithm for assessment of balloon positioning and filling. It must be stressed that aiming for a specific range of acceptability of the ratio between when ΔP_es_ and ΔP_aw_ corresponds mathematically to the introduction of a systematic percent error of 10 to 20%. This has important implications especially in patients in which the calibration was performed with small changes in the ΔP_es_, namely those that are spontaneously breathing with a limited inspiratory effort or those in controlled ventilation in which chest compressions resulted in small changes of the ΔP_es_. In these subjects, while during the calibration the absolute differences between ΔP_es_ and ΔP_aw_ are limited, a 10–20% percent error may result in large absolute errors when high inspiratory pressures or elevated inspiratory efforts are generated during tidal breathing.

Occlusion test and external chest compression test to verify inflation and positioning **Pros:** Simple and established method for verifying positioning and inflation of the balloon.

**Cons:** Balloon misplacement could be difficult to assess especially in passive conditions, where the magnitude of balloon pressure swings could suggest correct positioning also when the balloon is not in the distal third of the esophagus. In active patients, inspiratory efforts may be irregular, making titration of balloon inflation difficult.

## Interpretation of esophageal pressure in controlled ventilation

Once ensured the correct positioning and inflation of the esophageal balloon, further reasoning and computations are necessary to use it as a tool to titrate mechanical ventilation settings.

### Absolute values and partitioning of respiratory mechanics

**Fig. 3:**
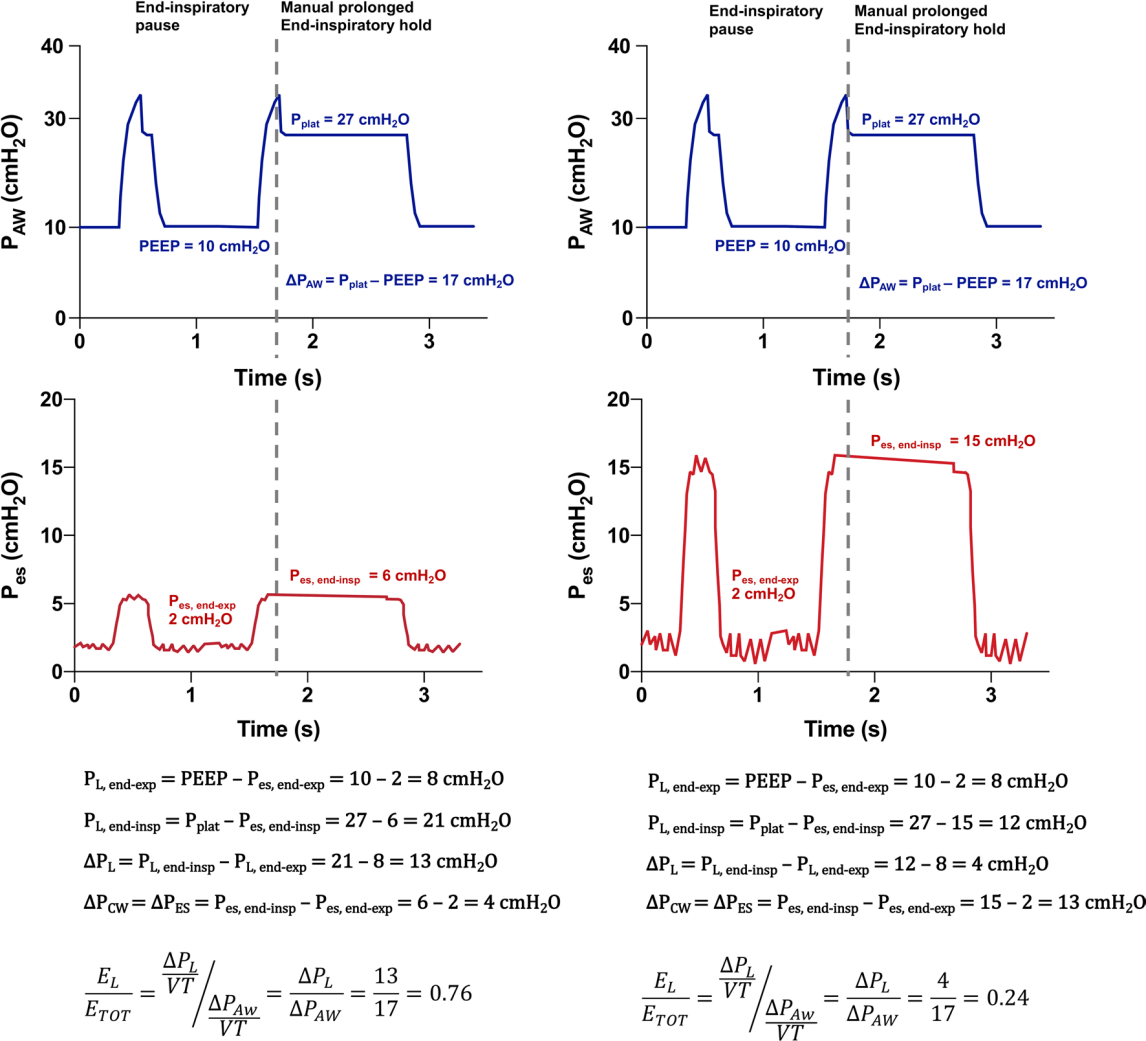
Airway pressure (top panels) and esophageal pressure (lower panels) of a patient with normal (left) or increased (right) chest wall elastance. At the same increased airway plateau and driving pressures (27 and 17 cmH_2_O, respectively), the resulting transpulmonary pressure at end-inspiration and the transpulmonary driving pressure is lower in the patient with increased chest wall elastance. The elastance ratio is reported, showing that in the left patient 76% of the total elastance is constituted by lung elastance, while only 24% in the patient on the right. P_AW_: airway pressure; P_es_: esophageal pressure; P_CW_: pressure of the chest wall; P_L_: transpulmonary pressure; P_plat_: plateau pressure; PEEP: positive end-expiratory pressure

The simplest application of esophageal pressure monitoring in passive mechanically ventilated patients is the partitioning of the respiratory system elastance (E_rs_) in its two components: lung elastance (E_L_) and chest wall elastance (E_cw_) [1, 3, 20]. Elastance is defined as the ratio between pressure changes and volume changes, is measured in cmH_2_O/L and is the reciprocal of compliance (C), thus E = 1/C. Elastance has additive properties, therefore E_rs =_ E_L_ + E_cw_ and since volume changes of the lungs are reflected by equal volume changes of the chest wall due to their anatomical contiguity, such property translates to the driving pressure (ΔP), namely the difference between end-inspiratory and end-expiratory pressure during tidal breathing. Therefore ΔP_rs =_ ΔP_L_ + ΔP_cw_, where ΔP_cw_ equals the driving esophageal pressure (ΔP_es_). As illustrated in Fig. 3, at equal plateau pressures measured at the ventilator, a patient with increased E_cw_ will have lower end-inspiratory transpulmonary pressure and correspondingly a lower lung strain. This is the pathophysiological basis of the concept that higher airway plateau pressures could be tolerated in case of increased E_cw_, such as in obese patients [21] or those with intraabdominal hypertension [22].

Partitioning of lung and chest wall elastance based on esophageal pressure **Pros:** Esophageal pressure monitoring allows partitioning of total respiratory system mechanics in its pulmonary and chest wall components.

**Cons:** There is limited consensus on safe upper limits of end-inspiratory and tidal driving transpulmonary pressures. End-inspiratory transpulmonary pressures below 15–20 cmH_2_O and tidal driving pressures below 10–12 cmH_2_O may be acceptable in ARDS. Conventional partitioning does not account for regional differences in pleural pressure.

### Elastance-derived interpretation

The elastance-derived method proposes to use the ratio of the lung elastance to the total elastance (elastance ratio, E_L_/E_rs_) as a multiplicative correction factor to apply to pressures measured at the ventilator (Fig. 3) [23]. The E_L_/E_rs_ ratio can be measured as (ΔP_rs_ − ΔP_es_)/ΔP_rs_ under passive conditions, and typically ranges from 0.5 or lower to 0.9 in critically ill patients with ARDS. It can be seen as the fraction of the airway pressure that is transmitted to the lungs. According to this method, inspiratory transpulmonary pressure is corrected as P_plat,elastance-derived_ = P_plat_ ⨉ E_L_/E_rs_ and has been shown to reflect accurately the regional transpulmonary pressure in the non-dependent regions [9]. This method has been extensively used by some research groups [24]; however, when used as guidance to set positive end-expiratory pressure (PEEP) it has poor agreement with methods relying on the absolute values of P_es_ [25].

Elastance-derived interpretation of transpulmonary pressure **Pros:** The elastance-derived interpretation of transpulmonary pressure provides an estimate of how the inspiratory pressure is partitioned between lung and chest wall in passive patients.

**Cons:** This method tends to reflect the elastic properties of the ventral lung, with limited information on the dependent dorsal regions.

### PEEP-release method

**Fig. 4:**
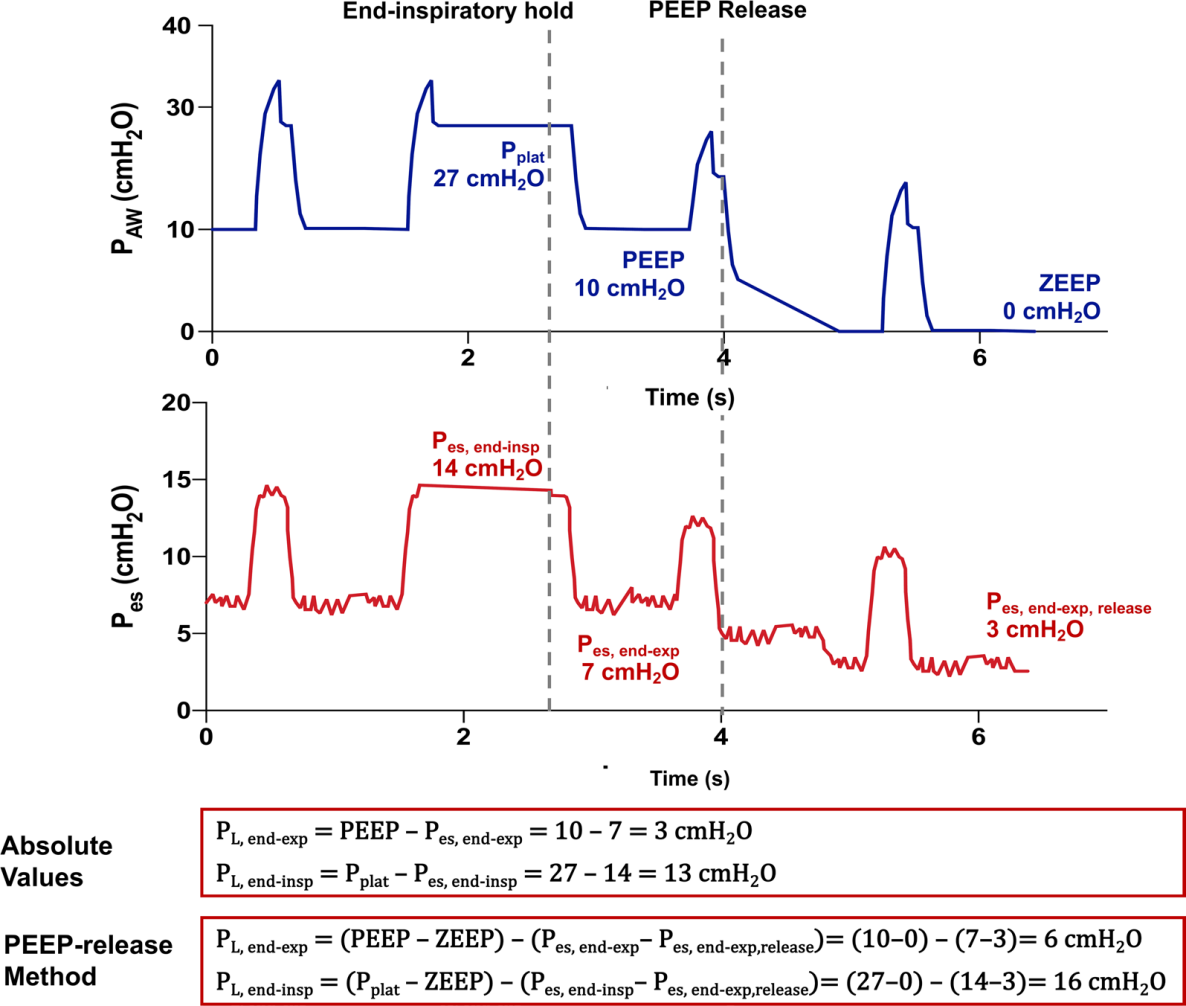
Comparison between transpulmonary pressure computed based on absolute values and using the PEEP-release method. From an initial PEEP of 10 cmH_2_O a peep-release maneuver is performed to measure the value of the end-expiratory esophageal pressure at ZEEP. P_AW_: airway pressure; P_es_: esophageal pressure; P_L_: transpulmonary pressure; P_plat_: plateau pressure; PEEP: positive end-expiratory pressure; ZEEP: zero end-expiratory pressure

To avoid the possible confounding factor of PEEP on the elastic properties of the chest wall, the PEEP-release method was proposed, based on the comparison of the transpulmonary pressure values during tidal breathing at PEEP with those obtained at zero end-expiratory pressure [2, 26]. Details on this calculation are provided in Fig. 4; as for the elastance-derived method, there is poor agreement between the values obtained with this method and those relying on absolute values of P_es_.

PEEP-release interpretation of transpulmonary pressure **Pros:** This method could allow measuring transpulmonary pressure avoiding the effect of PEEP on the chest wall.

**Cons:** This method is complex, has limited acceptance and requires acquiring respiratory mechanics data at PEEP of 0 cmH_2_O, a procedure raising safety concerns in severely hypoxemic patients.

### PEEP-step method

A research group proposed a method to estimate the transpulmonary pressure without an esophageal balloon, based on the measurement of the end-expiratory lung volume changes following an abrupt change in PEEP [27]. This method has been validated in an in-vitro model [28], but assumes implicitly that the end-expiratory transpulmonary pressure estimated with esophageal manometry is zero regardless of the applied PEEP level, which is contradicted by other clinical studies [29, 30].

PEEP-step method estimation of transpulmonary pressure **Pros:** This method could allow estimating transpulmonary pressure without an esophageal balloon.

**Cons:** Limited validation and clinical acceptance.

### Regional variability of pleural pressure and application of correction factors

**Fig. 5:**
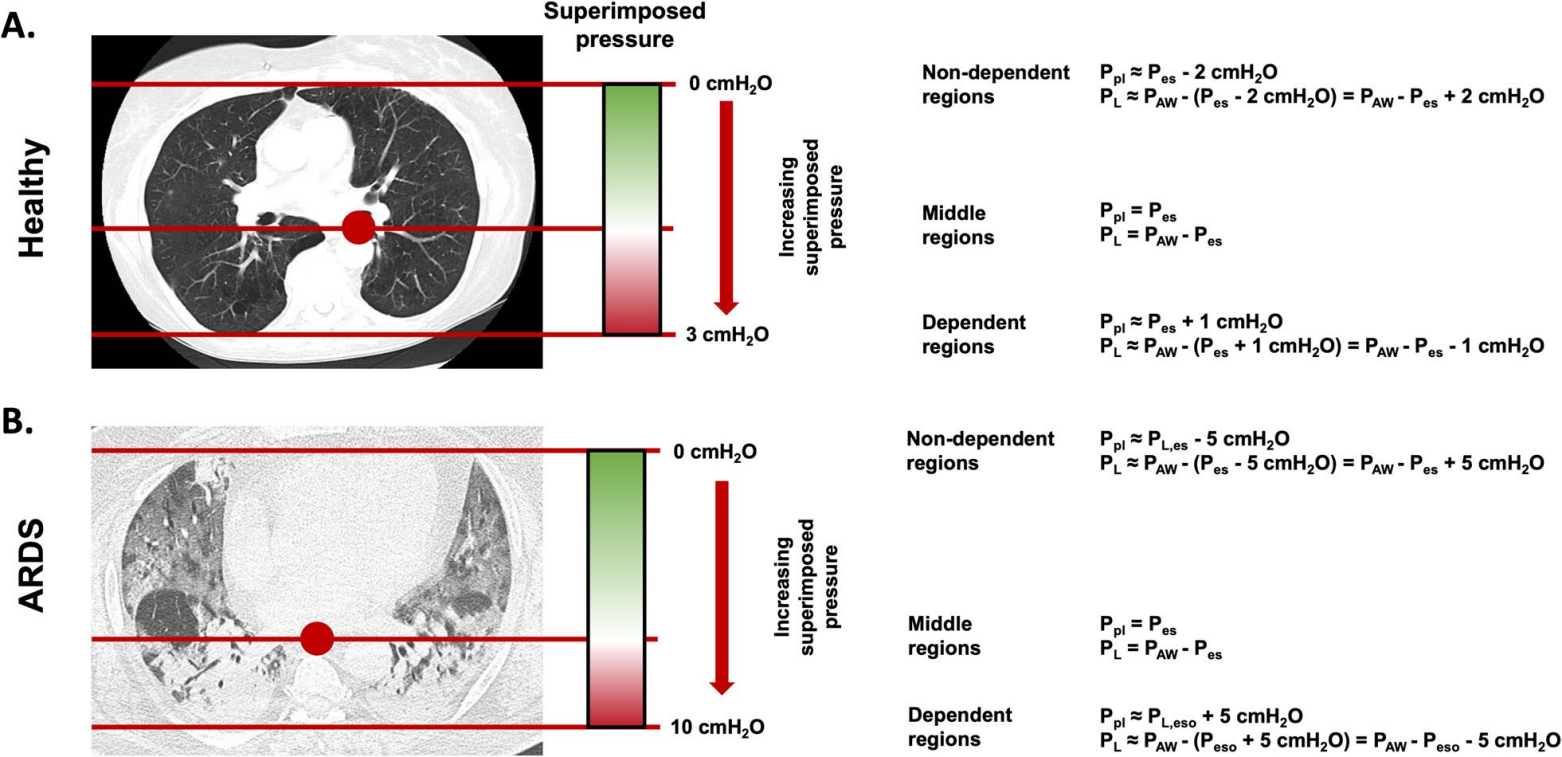
Contribution of the superimposed pressure to the esophageal pressure in a healthy patient (**A**) and in one with ARDS (**B**). Esophageal pressure under-estimates the transpulmonary pressure in non-dependent regions while over-estimates that in the dorsal ones, especially in injured lungs. P_AW_: airway pressure; P_es_: esophageal pressure; P_L_: transpulmonary pressure; P_plat_: plateau pressure

An important determinant of the P_pl_ at the regional level is the presence of the hydrostatic pressure due to the weight of lung tissue and mediastinum lying above the level at which P_pl_ is measured [31, 32]. This results in a ventral to dorsal gradient of the P_pl_ in the supine position; as a consequence, P_es_ approximates accurately the P_pl_ only measured at the level corresponding to the position of the esophagus in the chest wall [9]. As illustrated in Fig. 5A, in healthy lungs the superimposed pressure in the most dorsal regions is around 3 cmH_2_O [33], therefore assuming that the esophagus lies in an intermediate position in the ventral-dorsal axis, the P_pl_ in the most ventral or dorsal regions could deviate from the measured P_es_ by ± 1.5 cmH_2_O, a negligible value in most clinical settings. In ARDS, the weight of the injured lungs increases this gradient when fully supine to an average value of 10 cmH_2_O [31] (Fig. 5B), therefore P_pl_, _dorsal_ ≈ P_es_ + 5 cmH_2_O and P_pl,ventral_ ≈ P_es_ − 5 cmH_2_O [9]. The superimposed pressure in ARDS is therefore in the same order of magnitude of pressure changes applied to titrate mechanical ventilation at the bedside, notably PEEP. This has practical consequences when using P_es_ to guide clinical decisions. In fact, titrating mechanical ventilation parameters including PEEP using the P_es_ as estimate of the average P_L_ is equivalent to targeting the middle regions of the lungs. This may lead to airway pressures insufficient to fully recruit dorsal regions, but still resulting in hyperdistension in ventral regions.

Use of correction factors on P_es_ to estimate regional P_pl_ **Pros:** The application of correction factors of ± 5 cmH_2_O may provide an estimate of regional P_pl_ in ARDS, to allow separate assessment of P_L_ in lung regions at risk of de-recruitment versus those at risk of barotrauma.

**Cons:** Correction factors complicate substantially the interpretation of P_es_. Titrating ventilation settings based on the uncorrected value of P_es_ already represents a compromise between the risk of dorsal lung de-recruitment and ventral hyper-distension.

### PEEP titration based on end-expiratory transpulmonary pressure

The absolute end-expiratory transpulmonary pressure, when P_es_ is assumed equal to P_pl_, is P_L,end-exp_ = PEEP_tot_ − P_es,end-exp_. Its value in patients with ARDS typically ranges between − 10 to + 10 cmH_2_O and is influenced by PEEP [34] and positioning [35]. Negative values of P_L,end-exp_ are associated with de-recruitment in dependent lung regions, as confirmed in studies based on electrical impedance tomography (EIT) [36]. A first randomized trial comparing a PEEP titration strategy aimed at maintaining strictly non-negative P_L,end-exp_ showed improvement of oxygenation compared to a conventional low-PEEP/FiO_2_ table strategy [30]. However, this strategy was not superior to the conventional high-PEEP/FiO_2_ table in a larger randomized trial [37]. Nonetheless, a sub-study of the latter trial identified that titration of PEEP to P_L,end-exp_ to near-zero values (± 2 cmH_2_O) was associated with improved mortality, whereas higher values could result in high static strain and higher mortality [38].

PEEP titration based on end-expiratory transpulmonary pressure **Pros:** This approach may help identifying patients with relevant amount of de-recruited lung tissue and to individualize PEEP setting.

**Cons:** Randomized trials did not show clear mortality benefits. Increasing PEEP to excessively positive P_L,end-exp_ could be associated with worse outcome.

### Use of transpulmonary pressure to assess the risk of VILI

Exposure of lung regions to excessively elevated inspiratory pressures is a major determinant of ventilator-induced lung injury (VILI) [39]. This risk is higher in non-dependent ventral regions that receive most ventilation in ARDS in the supine position during controlled ventilation in passive patients. At end-inspiration, the P_L_ in the ventral lung is correctly estimated by the elastance-derived method or applying a correction of + 5 cmH_2_O on the absolute measurement of P_L_ [9]; however, also the application of a fixed correction factor is simplistic, as its exact value depends on the severity of ARDS. To assess dynamic strain, the transpulmonary driving pressure (ΔP_L_) could be used, computed as P_L,end-insp_ minus P_L,end-exp_.

Assessment of risk of VILI using transpulmonary pressure **Pros:** Limiting inspiratory and driving transpulmonary pressure could protect the lungs from excessive stress and strain. The elastance-derived method or the application of a correction factor of + 5 cmH_2_O on absolute measurements reflects the stress applied to the ventral regions.

**Cons:** Lack of consensus on safety thresholds.

## Interpretation of esophageal pressure in assisted ventilation

**Fig. 6:**
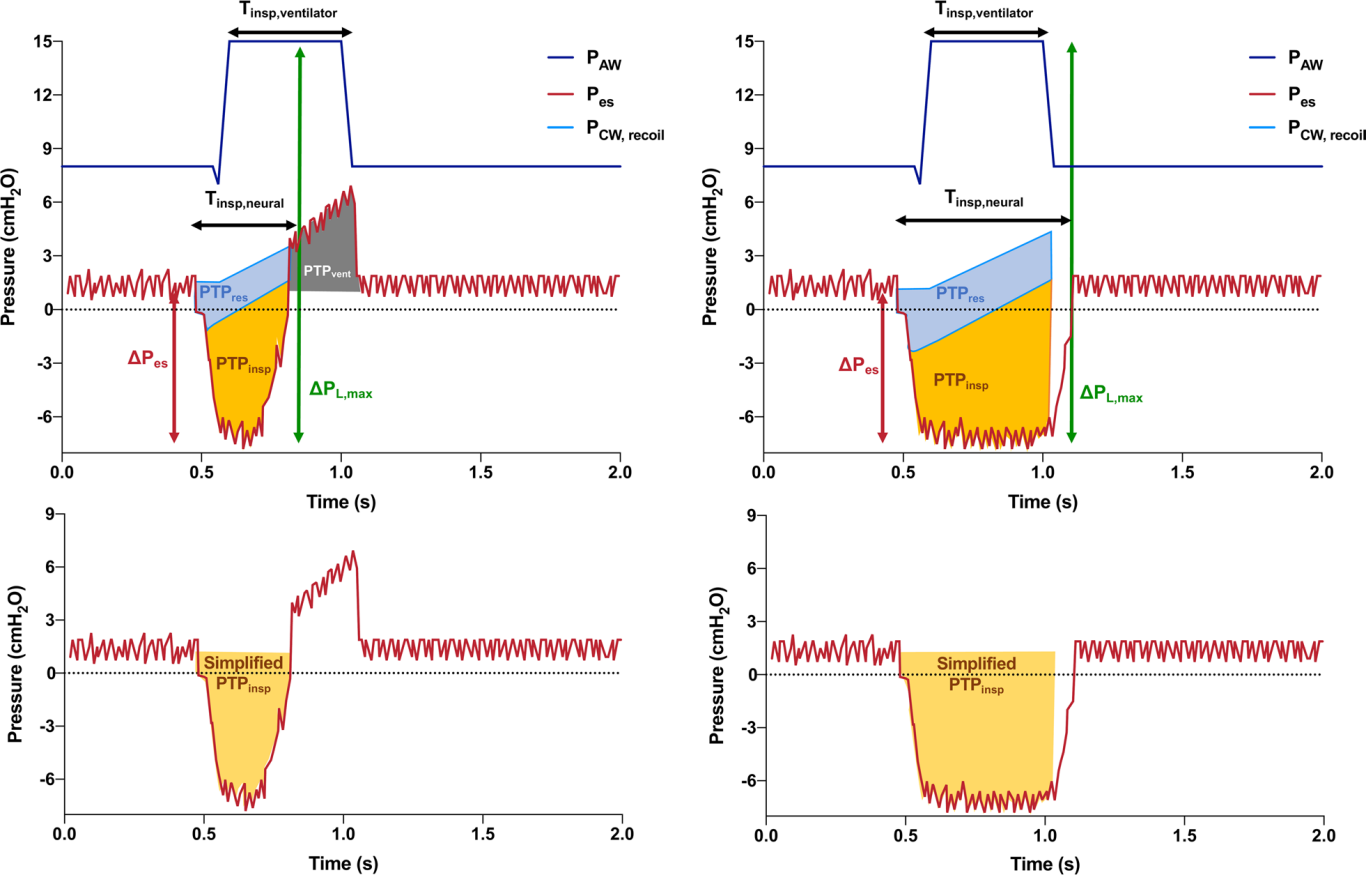
Computation of the pressure–time product (PTP) in two patients with equal magnitude of esophageal pressure swings (ΔP_es_) but short (left) versus prolonged (right) neural inspiratory time. When the inspiratory effort is initiated, the patient has first to overcome the intrinsic PEEP (PTP_res_, blue area, top panels), then the negative pressure is maintained for the duration of the neural inspiratory time (PTP_insp_, yellow regions, top panels). The patient on the right, compared to that on the left, has an higher PTP at the same ΔP_es_. If the inspiratory effort is terminated before the end of the ventilator inspiratory time, the patient acts as a passive patient in the late phase of inspiration, when the esophageal pressure increases because of the ventilator inspiratory pressure (PTP_vent_). The slope of the recoil pressure is chest wall elastance; when this is not known, PTP can be approximated integrating the P_es_ instead of the P_musc_ (simplified PTP_insp_, lower panels). P_AW_: airway pressure; P_es_: esophageal pressure; P_L_: transpulmonary pressure; P_CW,recoil_: pressure of the chest wall under passive conditions; PEEP: positive end-expiratory pressure

The applications of esophageal pressure monitoring in actively breathing patients receiving invasive assisted ventilation require separate considerations. The activation of inspiratory muscles generates a negative deflection of the P_pl_, the magnitude of this deflection is referred to as ΔP_es_ (Fig. 6). This negative pressure is maintained for a certain amount of time, that is the neural inspiratory time, and initially is spent to activate the ventilator’s inspiratory trigger and to overcome intrinsic (auto) PEEP and inspiratory resistive forces, then it is released to allow end of inspiration and cycling. The pressure generated by inspiratory muscles is defined as ${P}_{mus}={P}_{CW,recoil}-{P}_{es}$, where P_cw,recoil_ represents the pressure that would have been generated in the chest wall by the same gas volume in absence of inspiratory effort (Fig. 6, blue line). The maximum inspiratory transpulmonary pressure is the difference between the inspiratory airway pressure and the minimum P_es_ during the inspiratory effort (Fig. 6, green arrow). In case of increased inspiratory drive, very high P_L_ values could be reached when a highly negative P_es_ is added to the ventilator’s inspiratory pressure.

### Assessment of inspiratory effort and work of breathing

When titrating respiratory support in assisted ventilation modes, clinicians should ideally target the work of breathing (WOB): low WOB may reflect over-assistance or over-sedation, while higher WOB may indicate under-assistance or excessive respiratory effort and risk of patient self-inflicted lung injury [40]. Nonetheless, computation of WOB is complex, as it is defined as the area of the inspiratory portion of the inspiratory muscle pressure (P_mus_)—volume loop [3]. From a mathematical standpoint, this corresponds for each breath to:

$${WOB}_{breath}=\int\limits_{{T}_{insp}}{P}_{mus} dV$$

The WOB can be then expressed in Joules per liter of generated volume (${{WOB}_{volume}=WOB}_{breath}/{V}_{T}$) or per minute of ventilation (${{WOB}_{minute}=WOB}_{breath}\bullet RR$) [41]. A limitation of this definition of WOB is that any inspiratory effort not generating a tidal volume will be zero, leading to a misinterpretation of the role of ineffective inspiratory efforts and other asynchronies. Moreover, integration over a volume is a complex computation, therefore a surrogate based on integration over time is often used: the esophageal pressure–time product (${PTP}_{es}=\int_{{T}_{insp}}{P}_{mus} dt$, see Fig. 6). The PTP_es_ has been suggested to have a target range of 50 to 150 cmH_2_O·s [3] and can be calculated also for ineffective efforts. Still, computation of P_cw,recoil_ to obtain the P_mus_ requires knowing the E_CW_ (slope of the blue line in Fig. 6), which cannot be easily measured in active patients. Even if E_CW_ is measured in the same patient under passive conditions before initiation of assisted ventilation, it is unknown how E_CW_ varies when sedation is reduced, or neuromuscular blockade withheld to allow spontaneous breathing. Most experimental studies computed the P_cw,recoil_ based on either the predicted value of E_CW_ or assuming a fixed value of 5 cmH_2_O/L. The PTP_es_ and WOB are correlated and provide a precise quantification of the strength of inspiratory muscle activity, and PTP is well correlated to the metabolic cost of breathing, namely oxygen consumption [42]. The use of E_CW_ to account for the role of chest wall in inspiratory effort implies that the lung total volume is above the threshold point of the pressure–volume loop where the chest wall is in relaxation conditions [43]: this may not be the case in patients with respiratory failure with reduced total lung volume, thus questioning the routine use of the P_cw,recoil_ to measure the PTP in patients with ARDS. Thus, a further simplification consists in ignoring the P_cw,recoil_ when computing the PTP_es_ (simplified PTP_es,_ bottom panels in Fig. 6). Both the conventional and the simplified PTP are computed after off-line post-processing of respiratory tracings in the context of clinical research, with little to no application in the current clinical practice. The only measure that can be obtained in real-time at the bedside is the magnitude of esophageal pressure swings (ΔP_es_), which is a rough estimate of inspiratory effort. However, this could reflect inaccurately the WOB: the same ΔP_es_ will result in different PTP_es_ if applied for a short versus long neural inspiratory time (Fig. 6, left and right panels).

Esophageal pressure monitoring represents the reference method to measure of inspiratory muscle activity and driving transpulmonary inspiratory pressure. Due to its complexity, several alternative methods based on ventilator measurements not requiring the insertion of an esophageal balloon have been proposed to guide the level of respiratory assistance and weaning from mechanical ventilation. These include the airway occlusion pressure at 100 ms from onset of inspiration (P_01_) [44], end-inspiratory occlusion [45] and brief end-expiratory occlusion [46] tests.

Quantification of inspiratory activity based on transpulmonary pressure in active patients **Pros:** Monitoring P_es_ during assisted spontaneous breathing allows estimation of inspiratory muscles activity.

**Cons:** Except for ΔP_es_, measurements are complex. In several clinical scenarios, surrogates not requiring esophageal pressure monitoring could be used to identify patients with excessively high inspiratory activity.

### Assessment of maximum inspiratory transpulmonary pressure

Most research on transpulmonary pressure monitoring in assisted ventilation focused on the quantification of respiratory effort. However, high inspiratory transpulmonary pressures can be achieved also during assisted breathing [40, 47]. Figure 6 illustrates two patients receiving assisted ventilation highlighting the maximum P_L_ achieved during a respiratory cycle; since the end-expiratory P_L_ can be different from 0, this does not necessarily correspond to the sum of ΔP_es_ and ΔP_aw_ (Fig. 6, top panels). Also without esophageal pressure monitoring, an end-inspiratory occlusion performed in a cooperative patient under relaxation conditions could provide an indirect estimate of the maximum inspiratory P_L_ during tidal breathing [45].

Monitoring inspiratory transpulmonary pressure in active patients **Pros:** Limiting inspiratory transpulmonary pressure could protect the lungs during assisted breathing.

**Cons:** Lack of established thresholds of safe P_L_ during assisted spontaneous breathing.

### Assessment of asynchronies

Unintended interactions between the patient respiratory muscle activity and the ventilator are referred to as patient-ventilator asynchronies and are associated with worse clinical outcomes in critically ill patients [48], even though a causal link between asynchronies and mortality has not been established. Esophageal pressure monitoring allows precise identification of the matching between patient efforts and respiratory acts delivered by the ventilator. While P_es_ can be considered a reference method for detection of asynchronies, visual inspection of ventilator curves by experienced clinicians identifies correctly most asynchronies [49].

### Monitoring of asynchronies using esophageal pressure in active patients

**Pros:** Allows precise monitoring of all types of patient-ventilator asynchronies.

**Cons:** In most cases, asynchronies can be detected by visual inspection of flow-time and airway pressure–time curves on the ventilator.

## Applications during non-invasive respiratory support

The same considerations discussed in active patients also apply to those receiving non-invasive respiratory support such as conventional or high-flow oxygen therapy, continuous positive airway pressure (CPAP) and bilevel non-invasive ventilation (NIV). In these circumstances, high inspiratory drive may be associated with increased risk of barotrauma [40] and need for endotracheal intubation [50, 51]. Nonetheless, assessing inspiratory effort in these patients is particularly difficult. In patients receiving oxygen therapy few parameters in addition to clinical examination can give rough estimates of the inspiratory effort, including respiratory rate, level of dyspnea, diaphragm ultrasound and nasal pressure swings [52, 53]. In patients receiving positive-pressure respiratory support through a ventilator and non-invasive interfaces such as masks or helmets, occlusion-derived maneuvers on the ventilator typically give unreliable information on the inspiratory effort due to the confounding effect of the interface volume and compliance [54]. Monitoring P_es_ provides unique information in this setting, but this remains a largely underexplored field as clinicians tend to be reluctant in inserting an esophageal balloon in an awake hypoxemic patient [55].

Monitoring transpulmonary pressure during non-invasive respiratory support **Pros:** Measurement of inspiratory effort in a challenging clinical scenario where few alternatives are available.

**Cons:** Requires placement of an esophageal balloon in an awake, hypoxemic patient with related discomfort and potential risks.

## Conclusions

Transpulmonary pressure monitoring based on measurement of esophageal pressure substantially improved our knowledge of the pathophysiology and management of critically ill patients with respiratory failure. However, the physiology behind its interpretation is complex, and simplistic approaches have so far failed in enlarging the number of clinicians routinely using this technique. The availability of modern esophageal probes, ventilators and dedicated monitors makes this technique applicable in any modern intensive care unit. Despite the availability of alternative methods, the use of esophageal pressure monitoring should be encouraged in the clinical practice as it improves understanding of respiratory failure and personalization of mechanical ventilation in critically ill patients.

## Data Availability

Not applicable.
